# Reutericyclin producing *Lactobacillus reuteri* modulates development of fecal microbiota in weanling pigs

**DOI:** 10.3389/fmicb.2015.00762

**Published:** 2015-07-28

**Authors:** Yan Yang, Xin Zhao, Minh H. A. Le, Ruurd T. Zijlstra, Michael G. Gänzle

**Affiliations:** ^1^Department of Agricultural, Food and Nutritional Science, University of AlbertaEdmonton, AB, Canada; ^2^School of Food and Pharmaceutical Engineering, Hubei University of TechnologyWuhan, China

**Keywords:** enterterotoxigenic *Escherichia coli*, ETEC, pigs, feed fermentation, reutericyclin, exopolysaccharides, *Lactobacillus reuteri*, probiotic

## Abstract

*Lactobacillus reuteri* is used as probiotic culture in food and feed applications; however, strain specific properties of *L. reuteri* that mediate probiotic activity remain unknown. This study aimed to determine effects of feed fermentation with exopolysaccharide and reutericyclin producing *L. reuteri* on the transition of the gut microbiome of piglets after weaning. The reutericyclin and reuteran producing *L. reuteri* TMW1.656 was compared to the reutericyclin negative and levan producing *L. reuteri* LTH5794 and unfermented controls. Both strains were fermented at conditions supporting exopolysaccharide formation, or at conditions not supporting exopolysaccharide formation. Fecal microbiota were characterized by partial sequencing of 16S rRNA genes, and by quantitative PCR targeting clostridial toxins. The transition to solid food resulted in a transient increase of *Proteobacteria* to 12% of total bacteria, and increased bacterial diversity by increasing the abundance of anaerobic fiber fermenting *Firmicutes*. Three weeks after weaning, *Prevotella* and *Lactobacillus* were among the dominant bacterial genera. Feed fermentation with *L. reuteri* affected the abundance of few bacterial taxa and particularly reduced the abundance of *Enterobacteriaceae* (*P* < 0.05) when compared to unfermented controls. Reutericyclin producing *L. reuteri* increased the abundance of *Dialister* spp. and *Mitsuokella* spp. (*P* < 0.05) but did not influence the abundance of clostridial toxins in the feces. In conclusion, data on the contribution of specific metabolic activities of *L. reuteri* to probiotic activity will facilitate the strain selection for probiotic applications in food and feed.

## Introduction

*Lactobacillus reuteri* is a host-specific intestinal symbiont of humans and vertebrate animals (Walter, 2008; Frese et al., 2011). *L. reuteri* is commercially applied in cereal fermentations (Brandt, 2014) and as probiotic culture (Tuohy et al., 2003). Strain- or lineage specific metabolic traits of *L. reuteri* mediate host-specific colonization (Frese et al., 2014; Wilson et al., 2014), and contribute to its competitiveness in cereal fermentations and improved bread quality (Galle et al., 2012; Lin and Gänzle, 2014; Zhao et al., 2015). Strain specific properties of *L. reuteri* that mediate probiotic activity, however, remain unknown.

Metabolic traits that were suggested to mediate probiotic activity of *L. reuteri* include acid resistance (Teixeira et al., 2014), histamine decarboxylation (Spinler et al., 2014), exopolysaccharide production (Chen et al., 2014), and antimicrobial activity against pathogens (Gänzle, 2004; Rea et al., 2014). Reuteran and levan from *L. reuteri* prevented adhesion of enterotoxigenic *Escherichia coli* (ETEC) *in vitro*, and reduced mucosa-adherent ETEC in a swine model (Wang et al., 2010; Chen et al., 2014). The production of reuterin and reutericyclin by *L. reuteri* were proposed to provide protection against *Salmonella* Typhimurium and *Clostridium difficile*, respectively (Hurdle et al., 2011; De Weirdt et al., 2012). However, only two studies performed in rodent models demonstrate that antimicrobial compounds from lactic acid bacteria are active *in vivo*. Bacteriocin production by probiotic lactic acid bacteria reduced infection by *Listeria monocytogenes* (Corr et al., 2007), and reduced colonization by vancomycin resistant enterococci (Millette et al., 2008). Evidence for activity of antimicrobial metabolites of probiotics against autochtonous microbiota is inconclusive. The bacteriocin producing *L. salivarius* Abp118 altered the gut microbiome of swine and mice when compared to controls that did not receive probiotics. Changes induced by the bacteriocin producing strain, however, were not different from those induced by a bacteriocin-negative derivative of the same strain (Riboulet-Bisson et al., 2012).

Probiotic applications of *L. reuteri* and related organisms specifically targeted piglets (Konstantinov et al., 2008). Gut microbiota of pigs undergo a transition after weaning (Konstantinov et al., 2006; Lallès et al., 2007). The microbiome of suckling pigs is dominated by lactobacilli (Konstantinov et al., 2006) while strict anaerobic *Firmicutes* and *Bacteroidetes* dominate the microbiome of adult pigs (Lamendella et al., 2011; Riboulet-Bisson et al., 2012). The abundance of lactobacilli decreases after weaning (Konstantinov et al., 2006), providing opportunity for overgrowth of pathogens. Probiotics may decrease the abundance of pathogens (Zhang et al., 2010; Bednorz et al., 2013). Supplementation with *L. amylovorus* reduced levels of ETEC in the intestine but did not alter the hindgut microbiome (Konstantinov et al., 2008; Su et al., 2008).

This study aimed to determine the effect of feed fermentation with *L. reuteri* on the development of the gut microbiome in weanling piglets. The experimental design aimed to determine the contribution of viable *L. reuteri*, exopolysaccharide formation by *L. reuteri*, and reutericyclin formation by *L. reuteri* on the evolution of the gut microbiome in weanling piglets. The fecal microbiome was characterized by high throughput sequencing of 16S rRNA genes, and by quantitative PCR (qPCR) specifically targeting *C. difficile, C. perfringens*, and toxins produced by these organisms.

## Materials and methods

### Feed fermentation and diet preparation

Wheat flour was provided by University of Alberta Swine Research and Technology Centre, mixed with an equal amount of tap water, and inoculated with approximately 10^7^ CFU g^−1^ of the levan-producing *L. reuteri* LTH5794 or the reuteran-producing *L. reuteri* TMW1.656. A more detailed account of the feed fermentation and the control experiments ensuring the identity of fermentation microbiota with the inoculum is provided by Yang et al. (2015). Feed fermentation was carried out with addition of 10% (w/w flour) sucrose to support levan or reuteran formation during fermentation, or addition of 5% (w/w flour) glucose and 5% (w/w flour) fructose, which do not support reuteran or levan formation by *L. reuteri* but result in a formation of comparable levels of lactic and acetic acids. A chemically acidified control was prepared with 5% (w/w flour) fructose, 5% glucose (w/w flour), and addition of lactic acid (80%) and glacial acidic acid in a ratio of 4:1 (v/v) to acidify the feed to a pH of 3.8 (Table 1). Basal diets were mixed with 20–50% fermented or acidified wheat to produce the experimental feeds. Control diet was obtained by a mixture of basal diet and unfermented wheat. All diets were formulated to meet or exceed nutrient recommendation of National Research Council Canada (NRC) (2012) for 5–10 kg pigs. Titanium dioxide (TiO_2_) was added to each of the test diets as an indigestible marker.

**Table 1:** **Experimental diets used in this study**.

**Components**	**Control**	**Chem. acid**	***L. reuteri***			
			**TMW1.656**		**LTH 5794**	
			**Sucrose**	**Glu + Fru**	**Sucrose**	**Glu + Fru**
Acids	–	+	+	+	+	+
*L. reuteri*	–	–	+	+	+	+
Reutericyclin	–	–	+	+	–	–
Reuteran	–	–	+	–	–	–
Levan	–	–	–	–	+	–

*+ means component present in the diet, − means component not present in the diet*.

### Animals and experimental design

This animal trial was approved by the University of Alberta Animal Care and Use Committee under the guidelines of the Canadian Council on Animal Care and was conducted at the University of Alberta Swine Research and Technology Centre, Edmonton, AB, Canada. A total of 36 crossbred castrated weaning male pigs (~21 d of age) were selected and housed in a temperature-controlled room (28 ± 2.5°C). Pigs were divided into six consecutive and similar blocks with six pigs per block and one pig per pen (0.5 × 1.22 m). The experiment was designed to indicate whether the bacterial metabolites lactic and acetic acid, reuteran, levan, or reutericyclin influence the evolution of gut microbiota (Table 1). One pig per block was assigned to one of the six diets for a total of six observations per diet (Yang et al., 2015). Pigs were offered *ad libitum* food and water intake allowing for adequate growth. Meals in mash form were provided in equal amount twice daily (at 8 a.m. and 4 p.m.). Fresh fecal samples were collected from the pen floor in a sterile plastic bag at weaning, and 1, 2, or 3 weeks after weaning. A total of 137 samples obtained were collected and stored at −20°C. Frozen samples were thawed, mixed aseptically by spatula and 2–3 g subsamples were stored at −80°C.

### DNA extraction

Bacterial DNA was extracted from fecal samples using QIAamp® DNA stool Mini kit (50) (Qiagen, Inc., Valencia, CA, USA), following the manufacturer's instructions. Fecal DNA was quantified by Nano-Drop spectrophotometer system ND-1000 (Thermo Fisher Scientific Inc., Wilmington, USA). DNA quality was assessed by determining the ratio of absorbance at 260 and 280 nm. Only DNA samples that had 260:280 nm ratios higher than 1.8 were used for further analysis.

### PCR primers and probes

Primers and probes used in this study are listed in Table 2. Sequences of genes coding for clostridial toxins (α-, β-, β2-, entero-, and ι-toxin) collected from GeneBank (http://www.ncbi.nlm.nih.gov/genbank). The sequence data were aligned with CLUSTAL-W (Thompson et al., 1994) to identify conserved sequences. Primers and probes were designed to target the conserved toxin sequences. The specificity of primer sequences was checked by Basic Local Alignment Search Tool (BLAST) (http://www.ncbi.nlm.nih.gov/blast/Blast.cgi). Primers and probes were synthesized by Integrated DNA Technologies (Coralville, IA, U.S.A.).

**Table 2:** **Oligonucleotide sequences of the primers and probes used in this study**.

**Target group/gene**	**Primer/probe**	Oligonucleotide sequence (5′−3′)^*^	**T_A_ (°C)^a^**	**Product size (bp)**	**References**
*Clostridium* cluster I	CI F	GTGAAATGCGTAGAGATTAGGAA	58	665	Le Bourhis et al., 2005
	CI R	GATYYGCGATTACTAGYAACTC			
*Clostridium* cluster XI	CXI F	ACGGTACTTGAGGAGGA	58	139	Schwab et al., 2011, this study
	CXI R	GAGCCGTAGCCTTTCACT			
Total bacteria	HDA F	ACTCCTACGGGAGGCAGCAGT	62	198	Walter et al., 2000
	HDA R	GTATTACCGCGGCTGCTGGCAC			
*C. perfringens* α toxin (*cpa*)	CPα F	CTTGGAGAGGCTATGCACTATTT	60	90	This study
	CPα P	6FAM-CCATATCATCCTGCTAATGTTACTGCCGT-TAMRA			
	CPα R	CTTAACATGTCCTGCGCTATCA			
*C. perfringens* β toxin (*cpb*)	CPβ F	TCAAACAACCCTGTATATGGAAATG	60	149	This study
	CPβ P	6FAM-ACGGAAGATATACTAATGTTCCTGCAACTG-TAMRA			
	CPβ R	GGAGCAGTTAGAACTACAGACAT			
*C. perfringens* β-2 toxin (*cpb2*)	CPβ2 F	TGCAACTTCAGGTTCAAGAGA	60	121	This study
	CPβ2 P	6FAM-ACCATTTGAGAAGCTTTAACATCATCTCCC-TAMRA			
	CPβ2 R	TTGTCTAGCAGAATCAGGGTTT			
*C. perfringens* enterotoxin (*cpe*)	CPe F	AGCTGCTGCTACAGAAAGATTA	60	101	This study
	CPe P	6FAM-CTGATGCATTAAACTCAAATCCAGCTGGT-TAMRA			
	CPe R	GAGTCCAAGGGTATGAGTTAGAAG			
*C. perfringens* ι toxin (*iap*)	CPia F	CGTGGAGGATATACCGCAAT	60	116	This study
	CPia P	6FAM-TGGTCCTTTAAATAATCCTAATCCA-TAMRA			
	CPia R	GGTGTGAGCTTTAATGCGTTT			
*C. perfringens* ε toxin (*etx*)	CP etx F	AGCTTTTCCTAGGGATGGTTA	58	112	Messelhäußer et al., 2007
	CP etx R	AACTGCACTATAATTTCCTTTTCC			
*C. difficile* toxin B (*tcdB*)	CD tcdB F	GAAAGTCCAAGTTTACGCTCAAT	56	176	van den Berg et al., 2006
	CD tcdB P	6FAM-ACAGATGCAGCCAAAGTTGTTGAATT-TAMRA			
	CD tcdB R	GCTGCACCTAAACTTACACCA			

* *Y = T/C. F, Forward; R, Reverse; P, Probe*.

a *Annealing temperature*.

### Quantification of clostridia and their toxins by qPCR

Quantitative PCR (qPCR) was performed on a 7500 Fast Real-Time PCR System (Applied Biosystems, Foster City, CA, USA) using methodology described earlier (Metzler-Zebeli et al., 2010). To obtain positive controls for primers and probes targeting clostridial toxins, gBlocks® Gene Fragments were designed and synthesized by Integrated DNA Technologies. Standard curves for quantification of toxin genes were generated with 10-fold serial dilutions of purified PCR amplicons, which were amplified from gBlocks® Gene Fragments with the same primer pair and probe. For quantification of eubacteria and *Clostridium* clusters, standard curves were generated with amplicons that were amplified from serial dilutions of fecal DNA. The concentration of amplicons was determined by Nano-Drop spectrophotometer system ND-1000.

Fecal DNA was diluted to a concentration of 100 mg/L and analyzed in duplicate in a MicroAmp Fast Optical 96-well reaction plate sealed with MicroAmp Optical Adhesive Film (Applied Biosystems). Genes coding for 16S rRNA and the ε-toxin were amplified with the Quanti Fast SYBR Green master mix (Applied Biosystems). Taqman Fast master mix (Applied Biosystems) was used for detection of other toxins with probes. qPCR reaction contained 12.5 μL master mix, 4 μL of 10 μM primer solution in water, 2 μL of template DNA, and 6.5 μL nuclease-free water. Amplification of target sequences was achieved in 40 PCR cycles with primer annealing temperatures as shown in Table 2. Specific amplification of the target DNA was verified by melting curve analysis where applicable and by determination of the size of the amplicons by agarose gel electrophoresis.

### Sequencing of 16S rRNA sequence tags and sequence data analysis

High throughput sequencing of 16S rRNA sequence tags was performed by the University of Minnesota Genomics Center (Minneapolis, MN, USA) on a Illumina MiSeq. The V1–V3 regions of the 16S rRNA gene was amplified using primers Meta_V1_27F (TCGTCGGCA GCGTCAGATGTGTATAAGAGACAG **AGAGTTTGATCMTGGCTCAG**) and Meta_V3_534R (GTCTCGTGGGCTCGGAGATGTGTATAAGAGACAG **ATTACCGCGG CTGCTGG**). The bold part of each primer is complementary to the eukaryotic 16S sequences while upstream sequences corresponded to Illumina adapters that are required for sequencing and multiplexing. Paired-end sequencing was performed according to the manufacturer's instructions.

The QIIME pipeline (MacQIIME 1.8.0 20140103 OS10.6) (Caporaso et al., 2010) was used to analyze the sequences of 16S rRNA genes. PANDAseq (Masella et al., 2012) was used for quality filtering and assembly of the two ends of each read into contigs. Pairs with miscalled or uncalled bases in the overlapping region were discarded. Operational Taxonomic Units (OTUs) were generated using the UPARSE workflow (Edgar, 2013). Briefly, all sequences were merged into a single file, and the library name was used for multiplexing. To minimize computing time, sequences were dereplicated and sorted by abundance. Unique sequences in the data set were discarded. Sequences were clustered into OTUs by USEARCH (Edgar, 2010) using the Greengenes reference database (release October 2013), and a 97% similarity threshold. UCHIME (Edgar, 2010; Edgar et al., 2011) was used for filtering of chimeric sequences. OTUs with abundance below 0.005% of the total number of sequences were discarded (Bokulich et al., 2013).

Downstream analyses including taxonomy assignments, and alpha and beta diversity estimations were conducted using the QIIME workflow core_diversity_analysis.py, with a sampling depth of 7939 (Navas-Molina et al., 2013). This analysis was conducted with default parameters: taxonomy was assigned using Ribosomal Database Project (RDP) Classifier V2 (Wang et al., 2007), alpha diversity was estimated by Phylogenetic Diversity (PD) Whole Tree, Chao 1 and Observed Species indices (Colwell et al., 2012), beta-diversity was estimated through UniFrac distances (Vázquez-Baeza et al., 2013).

### Statistical methods

Data analyses of relative abundance and qPCR results were performed in SAS, (version 9.3, SAS Institute, 2012). The gene copy numbers of *Clostridium* cluster I and XI were converted into percentage of total bacteria gene counts for analysis. Mixed Procedure (Proc MIXED) was used based on randomized complete block design with repeated measurement. In the model, diet and week and diet × week were considered as fixed effects, while block was considered as random effect and pigs were considered as experimental unit. Comparisons of treatments were determined by contrast of target groups (SAS version 9.3). For relative abundance analysis, data obtained at weaning were used as covariate when comparing the difference between combination groups to assess the effects of *L. reuteri* and its metabolites exopolysaccharides and reutericyclin.

To test hypotheses, *p* < 0.05 was considered significant, after Bonferroni-adjustment. Normality of all variables was tested by Kolmogorov-Smirnoff test (Young, 1977). Results are presented as means ± standard deviation. Alpha- and beta- diversity were analyzed in MacQIIME v1.8.

## Results

### Effects of diet on animal health

All pigs remained healthy during the experiment period and diarrhea or any clinical signs of disease were not observed. Prior analyses of samples obtained in the same study reported the strain-specific quantification of *L. reuteri* and ETEC but not the overall composition of the hindgut microbiome (Yang et al., 2015).

### Diversity of the fecal microbiome of piglets

During the feeding trial, 137 fecal samples were collected and a total of 5,292,722 sequences with a minimum of 7939 sequences per sample were obtained. A total of 7434 OTUs were identified, representing 6 phyla, 27 families, 42 genera, and 49 species. Phyla in fecal samples and their abundance included *Bacteroidetes* (40.8–45.8%), *Firmicutes* (35.8–45.1%), *Proteobacteria* (0.9–12.9%)*, Tenericutes* (0.7–5.2%), *Spirochaetes* (1.4–3.1%), and *Planctomycetes* (0–1.3%).

**Figure 1:**
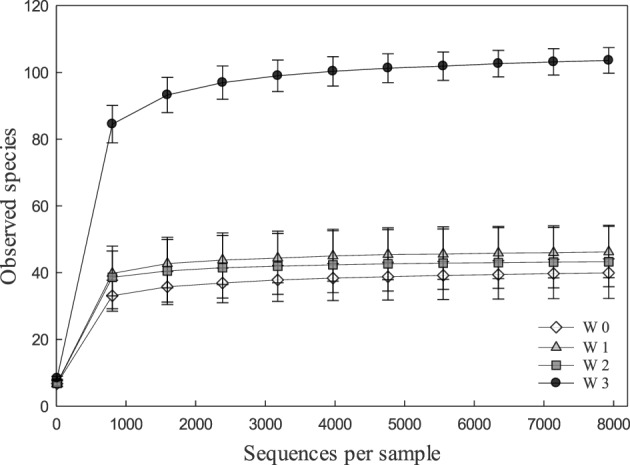
**Rarefaction curves indicating the effect of the number of partial sequences of 16S rRNA genes that were analyzed on the number of OUT's in fecal microbiota of pigs**. Rarefaction curves were calculated in QIIME with the sample depth of 7939 sequences per sample from 137 fecal samples obtained at weaning (week 0, *n* = 29), or at week 1 (*n* = 36), week 2 (*n* = 36), and week 3 (*n* = 36) after weaning.

**Figure 2:**
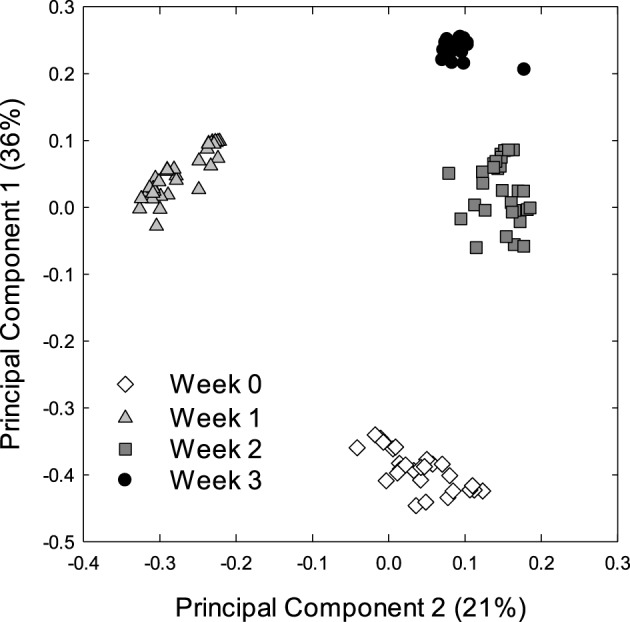
**Principle coordinate analysis (PCA) of the bacterial microbiota of piglets**. The PCA plot was generated using the unweighted UniFrac distance metric. Each dot represents an individual sample collected at the start of weaning week 0 (*n* = 29) or after week 1 (*n* = 36), week 2 (*n* = 36), and week 3 (*n* = 36) of weaning.

Alpha and beta diversity analyses revealed that the effects of treatments were small when compared to the differences occurring over time. In Alpha diversity metrics (within sample diversity), species richness of samples taken at week 3 was significantly higher (*p* < 0.001) than the diversity of samples taken earlier in the experiment (Figure 1) but differences between diets were not significant (*p* > 0.05). Beta diversity (between sample diversity) also demonstrated that samples taken at different times differed (*p* < 0.0001) (Figure 2). The distances between samples within each week were always smaller than the between week comparisons (Figure 2).

### Transition of bacteria over time and prevalent bacterial genera

**Figure 3:**
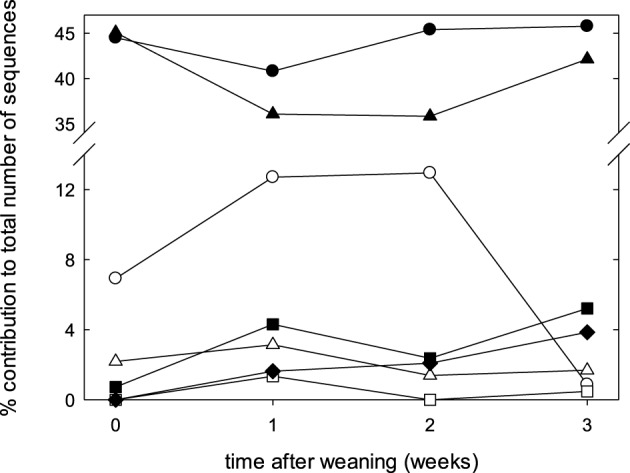
**Composition of fecal microbiota at the phylum level at weaning, and during the first 3 weeks after weaning**. Data represent the median proportions of each phylum as determined by the RDP (Ribosomal Database Project) classifier. The phyla are represented by symbols as follows: ●, *Bacteroidetes*; ▴, *Firmicutes*; 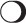, *Proteobacteria;* Δ, *Spirochaetes*; ■ *, Tenericutes*; 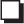, *Planctomycetes*; ♦, unassigned.

The fecal microbiome was characterized by analysis of the relative abundance of bacterial taxa at the phylum level (Figure 3) and at the genus level (Table 3). The proportion of *Bacteriodetes* and *Firmicutes* remained unchanged over the study period. Major shifts were observed in the *Proteobacteria, Tenericutes*, and *Planctomycetes* (Figure 3). *Proteobacteria* peaked at week 1 and 2 and decreased again at week 3; *Tenericutes* and *Planctomycetes* increased over time (Figure 3). Within the *Bacteroidetes, Prevotella* and the unassigned S24-7 genus increased while *Bacteroides* and other unknown genera decreased (Table 3). The overall increase of microbial diversity (Figure 1) was largely attributed to increased abundance and diversity of bacterial taxa in the *Firmicutes*. Changes in the *Proteobacteria* were mainly attributable to *Enterobacteriaceae* (compare Table 3 and Figure 3).

**Table 3:** **Relative abundance (%) of bacterial genera in fecal microbiota of pigs at weaning (week 0) and at week 1, 2, or 3 after weaning, determined by Illumina sequencing of 16S rRNA tags**.

**Genus**	**Week 0**	**Week 1**	**Week 2**	**Week 3**
***BACTEROIDETES***				
*Bacteroides*	13±2.6	ND	ND	ND
*Parabacteroides*	2.2±4.3^ab^	3.1±2.6^a^	1.7±0.8^b^	1.2±0.43^b^
*Prevotella*	8.1±0.71^a^	9.6±1.1^ab^	14±1.0^b^	20±2.8^c^
*[F: S24-7]*	3.9±2.6^a^	10±5.5^b^	15±6.9^c^	15±0.45^c^
*CF231*	ND	ND	ND	0.47±0.39
*[F: Paraprevotellaceae]*	ND	2.3±1.0^a^	0.6±0.07^b^	1.3±0.7^b^
*Other (O: Bacteroidales)*	11±22	ND	ND	ND
*[O: Bacteroidales]*	1.7±0.04^a^	ND	1.7±0.04^a^	3.2±1.5^b^
*[F: P-2534-18B5]*	5.4±0.01^a^	16±32^b^	14±10^b^	3.9±3.1^a^
***FIRMICUTES***				
*Lactobacillus*	8.2±15^a^	12±1.3^ab^	15±5.1^b^	11±11^ab^
*Other (O: Clostridiales)*	ND	1.1±0.2^a^	ND	0.2±0.04^a^
*[O: Clostridiales]*	4.0±3.6^ab^	4.4±11^a^	1.8±1.7^b^	6.5±4.1^a^
*[F: Christensenellaceae]*	2.0±0.14^a^	2.9±11^a^	1.6±1.2^a^	0.59±0.51^a^
*[F: Clostridiaceae]*	ND	ND	ND	0.46±0.02
*Blautia*	ND	ND	ND	0.47±0.08
*Coprococcus*	ND	0.9±1.7^a^	2.4±0.43^a^	0.86±0.57^a^
*Lachnospira*	ND	ND	1.1±0.16^a^	0.82±0.07^a^
*Roseburia*	1.3±1.7^a^	ND	0.3±0.02^b^	1.1±0.32^a^
*Other (F: Lachnospiraceae)*	2.6±0.32^a^	ND	0.47±0^b^	ND
*[F: Lachnospiraceae]*	3.8±1.6^a^	4.6±3.7^a^	2.2±1.6^b^	2.9±0.74^ab^
*Faecalibacterium*	ND	ND	1.0±0.29^a^	0.7±0.09^b^
*Oscillospira*	1.4±0.89^a^	ND	0.41±0.12^b^	1.5±0.22^a^
*[F: Ruminococcaceae]*	18±7.0^a^	6.7±0.4^b^	4.93±2.7^b^	5.7±0.75^b^
*Dialister*	ND	ND	ND	1.1±0.01
*Megasphaera*	ND	0.8±0.04^a^	2.6±0.01^ab^	3.7±0.03^b^
*Mitsuokella*	ND	ND	ND	1.1±0
*Bulleidia*	ND	ND	1.1±0.08^a^	0.84±0.11^a^
*Catenibacterium*	ND	ND	0.52±0^a^	0.46±0.06^a^
*Eubacterium*	0.48±0.28^a^	ND	ND	0.41±0.01^a^
*p-75-a5*	2.0±1.3^a^	1.6±1.5^a^	2.0±0.92^a^	1.1±0.66^a^
***PLANCTOMYCETES***				
*[F: Pirellulaceae]*	ND	1.4±0.2^a^	ND	0.5±0.68^b^
***PROTEOBACTERIA***				
*Desulfovibrio*	1.36±0.32	ND	ND	ND
*Succinivibrio*	ND	3.1±0.27^a^	ND	0.38±0.63^b^
*[F: Enterobacteriaceae]*	5.0±2.7^ab^	9.1±15^a^	9±16^a^	0.48±0.03^b^
***SPIROCHAETES***				
*Treponema*	2.1±0.85^ab^	3.5±1.2^a^	1.6±5.4^b^	1.9±0.52^b^
***TENERICUTES***				
*[O: RF39]*	1.1±0.26^a^	4.7±2.8^b^	2.3±1.4^a^	5.4±1.1^b^
Unassigned	ND	1.8±0.28^a^	2.3±0.36^a^	4.0±2.6^b^
Total	97.96	99.61	99.6	100

*Data are presented as means ± standard deviation of 29 (week 0) or 36 (weeks 1, 2, and 3) observations*.

*Data in the same row that do not share a common superscript are significantly different (P < 0.05). ND, not detected*.

*Unassigned genera are presented with upper level of family (F) or order (O) in square brackets. “Unassigned” means a good hit to a particular sequence, but that sequence has a poorly defined taxonomy itself at the genus level. “Other” means the assignment is ambiguous*.

### Clostridia clusters and toxins quantified by qPCR

To determine effects of feed fermentation on the *Clostridium* cluster I and XI, these organisms and toxins produced by *C. difficile* and *C. perfringens* were quantified using qPCR (Table 4 and data not shown). Both *Clostridium* clusters were relatively abundant in fecal samples; changes over time or changes within diets, however, were not significant (*p* > 0.05; data not shown). The α- and β-2 toxins from *C. perfringens* were detected in samples collected at week 0 and in a few samples from week 1 but not in samples taken at later times. Differences in the abundance of toxins in samples from animals fed different diets were not significant (*p* > 0.05). The abundance of other clostridial toxins, namely the β-, entero-, ι-, and ε-toxin of *C. perfringens* and the *C. difficile* toxin B, was below the detection limit of 3.6 log(copy number/g) in all samples (Table 4).

**Table 4:** **Quantification of alpha and beta-2 toxins of *C. perfringens* in fecal samples collected at weaning (week 0) and at week 1, 2, 3 after weaning**.

**Diets**	**α toxin log(copy number/g)**			**β-2 toxin log(copy number/g)**		
	**Week 0**	**Week 1**	**Weeks 2 and 3**	**Week 0**	**Week 1**	**Weeks 2 and 3**
Control	6.9 ± 1.4 (6/6)^a^	4.2 ± 0.5 (6/6)	ND^b^	6.2 ± 1.2 (6/6)	3.9 ± 0.5 (3/6)	ND
Chem. Acid	6.3 ± 0.8 (6/6)	3.8 ± 0.2 (2/6)	ND	5.6 ± 0.8 (6/6)	3.6 (1/6)	ND
TMW1.656 sucrose	6.6 ± 1.1 (4/4)	4.2 ± 0.3 (3/6)	ND	5.8 ± 0.9 (4/4)	3.7 ± 0.2 (3/6)	ND
TMW1.656 Glu+Fru	6.7 ± 1.1 (2/2)	3.9 ± 0.1 (4/6)	ND	5.6 ± 0.3 (2/2)	3.6 (1/6)	ND
LTH5794 sucrose	7.0 ± 0.4 (5/5)	4.2 ± 0.3 (4/6)	ND	6.3 ± 0.3 (5/5)	3.8 ± 0.1 (2/6)	ND
LTH5794 GluFru	6.6 ± 1.2 (5/5)	3.8 ± 0.5 (4/6)	ND	5.9 ± 1.2 (5/5)	3.8 ± 0 (2/6)	ND

*Data are presented as log(copy number/g feces) and reported as means ± standard deviation of positive samples*.

a *Data are reported as means ± standard deviation of positive samples. The number in brackets indicates the (number of positive samples/number of samples analyzed)*.

b *ND, not detected [below the detection limit of 3.6 log(copy number/g)]*.

### Effects of treatments on bacteria species

Analysis of diet-induced changes accounted for the individual differences between animals in the same group by using data from each pig at week 0 as covariate (Table 5). To analyse the effect of specific feed components or metabolites that were present in several diets, diets were grouped as follows: Diets containing *L. reuteri* (groups 3, 4, 5, and 6) or not (group 1 and 2); diets containing reutericyclin (groups 3 and 4) or not (groups 1, 2, 5, and 6), and diets containing exopolysaccharides (groups 3 and 5) or not (groups 1, 2, 4, and 6). Moreover, the impact of feed fermented with *L. reuteri* TMW1.656 was compared to *L. reuteri* LTH5794 (Table 5).

**Table 5:** **Effects of diets and reutericyclin producing *L. reuteri* on the composition of fecal microbiota of pigs 3 weeks after weaning, as determined by Illumina sequencing of 16S rRNA tags**.

**Variables**		**Effects of diets (relative abundance%)**					**Effects of L. reuteri (P-value)**		
**Species**	**Control**	**Chem. acidified**	**LTH5794 sucrose**	**LTH5794 Glu /+ Fru**	**THW1.656 sucrose**	**TMW1.656 Glu/+Fru**	**TMW1.656 vs LTH5794**	**TMW1.656 vs other diets**	***L. reuteri* vs no bacteria**
***BACTEROIDETES***									
[G: *Prevotella*]	6.63±2.99^B^	12.8±6.71^A^	6.23±2.22^B^	8.23±4.56^AB^	6.06±2.58^B^	5.05±3.3^B^	0.68	0.27	0.07
*Copri*	10.4±8.44^AB^	5.62±3.54^A^	14.2±5.88^AB^	8.59±6.46^AB^	16.9±7.38^B^	11.1±6.56^AB^	0.87	0.46	0.16
[F: *S24-7*]	19.4±7.42	14.0±1.47	16.4±2.64	16.5±3.27	11.7±3.93	13.88±6.1	0.09	0.06	0.22
[G: *CF231*]	0.48±0.31	0.95±0.43	0.22±0.19	0.46±0.46	0.3±0.23	0.38±0.21	0.96	0.31	0.02
[F: *p-2534-18B5*]	5.18±4.91	2.5±1.56	4.16±3.66	3.3±3.59	3.47±6.01	4.72±6.03	< 0.0001	< 0.0001	< 0.0001
***FIRMICUTES***									
[O: *Clostridiales*]	4.96±2.08	4.26±1.48	6.68±2.67	8.89±3.96	6.76±2.53	7.22±3.88	0.64	0.47	0.02
[G: *Coprococcus*]	0.14±0.25	0.2±0.3	1.52±1.4	1.21±1.73	1.36±3.25	0.73±0.65	0.049	0.35	0.07
[G: *Oscillospira*]	2.58±1.08	1.4±0.45	1.46±0.45	1.29±0.38	1.16±0.69	1.08±0.51	0.88	0.43	0.03
[G: *Dialister*]	0.19±0.37^A^	0.03±0.06^A^	0.02±0.03^A^	0.38±0.86^A^	4.37±7.08^B^	1.72±3.08^AB^	0.10	0.06	0.20
[G: *Mitsuokella*]	0.35±0.35^AB^	0.07±0.12^A^	0.16±0.2^A^	0.15±0.24^A^	3.74±5.43^C^	2.2±0.86^BC^	0.0001	< 0.0001	0.01
***PROTEOBACTERIA***									
[F: *Enterobacteriaceae*]	1.22±1.45	0.89±1.11	0.42±0.59	0.05±0.05	0.21±0.42	0.09±0.13	0.83	0.21	0.01

*Effects of diets were analyzed based on the relative abundance of bacteria groups. To analyse effects of reutericyclin producing L. reuteri, data obtained at weaning (week 0) of each pig sample are used as covariate when P-value was calculated. Data are presented as means ± standard deviation of 29 (week 0) or 36 (weeks 1, 2, and 3) observations and significant effects are shown*.

*Data in the same row that do not share a common superscript are significantly different (P < 0.05)*.

*Data indicating the effects of L. reuteri or reutericyclin are presented as P-value. P < 0.05 indicates statistically significant difference. ND means not detected. Unassigned species are presented as square brackets with upper level of genus (G), family (F), or order (O)*.

Significant differences between individual diets pertain to few bacterial taxa in the phyla *Bacteroidetes* and *Firmicutes* (Table 5). Any *L. reuteri* strain altered the abundance of 6 bacterial taxa when compared to the control diets (Table 5). These changes particularly included a reduced number of the family *Enterobacteriacae*. Diets containing reutericyclin significantly changed the abundance of a *Mitsuokella* species and a family in the phylum *Bacteroidetes* (Table 5); these differences were also significant when the reutericyclin negative strain *L. reuteri* LTH5794 was compared to the reutericyclin positive *L. reuteri* TMW1.656 (Table 5). The presence or absence of exopolysaccharides had no significant (*p* > 0.05) influence on any bacterial taxon.

## Discussion

The study investigated the effect of *L. reuteri* fermented diets on the development of intestinal microbiota of pigs after weaning. The experimental design aimed to determine the contribution of specific metabolites, i.e., reuteran, levan, and reutericyclin, on the evolution of the intestinal microbiome. The present study is the first employing high throughput sequencing to document the transition of the microbiome of piglets. In contrast, the development of the infant microbiota after birth is well documented (Koenig et al., 2011; La Rosa et al., 2014). The infant microbiome is characterized by low diversity and stability. Important determinants of the infant gut microbiome include the mode of delivery, type of feeding, antibiotic use, and the gestational age of the mother (Penders et al., 2006; Koenig et al., 2011). From birth to weaning, both human infants and piglets experience a succession of *Lactobacillus* spp. in the gut due to the consumption of milk (Tannock et al., 1990; Roger et al., 2010). The gradual adaptation of the diet in infants typically avoids major problems that are associated with a shifting microbiome. The sudden change of diet after weaning, however, often causes dysbiosis and diarrheal diseases in piglets, and is thus a major concern in pig production (Lallès et al., 2007).

*Bacteroidetes* and *Firmicutes* dominated the intestinal microbiome in piglets, in keeping with past studies on the swine microbiome (Leser et al., 2002; Lamendella et al., 2011; Riboulet-Bisson et al., 2012). The present study additionally documents that strict anaerobes such as *Ruminococcaceae, Bacteroides*, and *Prevotella* were the dominant bacteria at weaning. After weaning, *Lactobacillus* and *Prevotella* spp. replaced *Ruminococcaceae* and *Bacteroides* as the most abundant bacterial genera. Dietary changes modulate the gut microbiome of pigs (Lu et al., 2014). Bacteria belonging to *Bacteroides-Prevotella-Porphyromonas* play an important role in fiber degradation, and are stimulated by fermentable non-starch polysaccharides in pig diets (Metzler-Zebeli et al., 2010; Ivarsson et al., 2014). Human studies have linked the diversity of different plant fibers in whole grains to an increased diversity of the gut microbiome, particularly in the genera *Roseburia, Bifidobacterium, Eubacterium*, and *Dialister* (Martínez et al., 2013). Accordingly, low carbohydrate diets resulted in a substantial and diet-dependent reduction of *Firmicutes* (Duncan et al., 2008). The increase of bacterial diversity after weaning was particularly attributable to an increased diversity in the phylym *Firmicutes* and may thus be linked to the presence of whole wheat in the piglets' diet, which accounted for 20% of the diet after weaning and for 50% diet after week 1 (Yang et al., 2015).

The strains of *L. reuteri* used in the present study are rodent-lineage allochthones to the pig intestine (Su et al., 2012; Frese et al., 2014). Major changes in the intestinal microbiota were attributable to the presence of probiotic *L. reuteri* and its metabolites. The most prominent change attributable to the presence of *L. reuteri* was the reduced abundance of *Enterobacteriaceae*. This result conforms to prior reports obtained with diverse probiotic cultures (De Angelis et al., 2007; Konstantinov et al., 2008; Bednorz et al., 2013; Valdovska et al., 2014) and indicates that successful competition with *Enterobacteriaceae* is not a specific property of *L. reuteri*. Remarkably, this study also demonstrated that the abundance of several members of the *Firmicutes* and *Bacteroidetes* was influenced by *L. reuteri*.

Exopolysaccharides did not influence the composition of gut microbiota. Levan and reuteran are not digested by pancreatic digestive enzymes and selectively fermented by hindgut microbiota (Korakli et al., 2002; van Bueren et al., 2015). However, the exopolysaccharides levels in the feed used in this study, 1–3 g/kg feed (Yang et al., 2015), are low when compared to other studies reporting prebiotic intervention (Valdovska et al., 2014). Fermented feed containing reuteran, however, specifically reduced the abundance of enterotoxigenic *E. coli* (ETEC) in weanling piglets (Yang et al., 2015). The lack of any effect of reuteran on the overall composition of the gut microbiome (this study) coupled to the specific reduction of ETEC colonization (Yang et al., 2015) supports the hypothesis that effects of reuteran are mediated by a specific reduction of ETEC adhesion rather than a prebiotic effect (Chen et al., 2014).

Bioinformatic analyses of the metagenome of intestinal microbiota suggested that the ecology of colonic microbiota is shaped by competition for substrates rather than the production of antimicrobial compounds (Walter and Ley, 2011; Zheng et al., 2015). Accordingly, bacteriocin producing *L. salivarius* did not induce significant changes in the gut microbiome of pigs when compared to an isogenic bacteriocin-negative strain (Riboulet-Bisson et al., 2012). Medication of grower pigs with in feed antibiotics (Chlortetracycline, sulfamethazine, and penicillin), however, caused much more substantial changes of the colonic microbiome than was observed in this study for reutericyclin (Looft et al., 2014). Reutericyclin is a unique antimicrobial compound with broad spectrum of activity against Gram-positive bacteria (Gänzle et al., 2000; Gänzle, 2004; Hurdle et al., 2011; Lin et al., 2015). Reutericyclin is produced during growth of *L. reuteri* in wheat sourdough (Gänzle and Vogel, 2002), suggesting that feed fermentation with *L. reuteri* TMW1.656 delivers active concentrations of reutericyclin to the swine gut. We hypothesized that reutericyclin may reduce the abundance of the *Clostridium* clusters I and XI. The pathogenic species *C. perfringens* (cluster I) and *C. difficile* (cluster XI), are sensitive to reutericyclin (Hurdle et al., 2011; Hofstetter et al., 2013). However, neither the abundance of *Clostridium* cluster I and XI nor the abundance of genes coding for clostridial toxins was influenced by reutericyclin-producing *L. reuteri*. The abundance of *Dialister* and *Mitsuokella* increased upon feeding of the reutericyclin producing *L. reuteri* TMW1.656, possibly as a consequence of the inhibition of reutericyclin-sensitive competitors.

In conclusion, the present study monitored the transition of fecal microbiota of weanling piglets, and determined the impact of *L. reuteri* and its metabolites on this transition of intestinal microbiota. After weaning, bacterial diversity increased, mainly due to an increase of bacterial taxa in the phylum Firmicutes. Weaning was also associated by a transient increase of *Enterobacteriaceae*, which corresponds to the susceptibility of weanling piglets to infection by enteric pathogens. The gut microbiome of weanling piglets was not influenced by the inclusion of organic acids in the diet; however, the presence of viable *L. reuteri*, reutericyclin, and reuteran all affected the gut microbiome. Probiotic *L. reuteri* altered the abundance of several bacterial taxa, notably *Enterobacteriaceae* including *E. coli*. The reutericyclin producing strain significantly increased the abundance of two strict anaerobic members of the *Firmicutes*, while reuteran affected the colonization with ETEC but none of the numerically dominant members of fecal microbiota (Yang et al., 2015, this study). Data on the contribution of specific metabolic activities of *L. reuteri* to probiotic activity will facilitate the strain selection for probiotic feed applications in animal production. The study also novel opens novel avenues to reduce the incidence of childhood diarrhea in developing countries (Thapar and Sanderson, 2004) by application of probiotic cultures, or by food fermentations with probiotic *L. reuteri* (Sekwati-Monang and Gänzle, 2011).

### Conflict of interest statement

The authors declare that the research was conducted in the absence of any commercial or financial relationships that could be construed as a potential conflict of interest.
